# Localization-Specific Expression of CCR1 and CCR5 by Mast Cell Progenitors

**DOI:** 10.3389/fimmu.2020.00321

**Published:** 2020-02-26

**Authors:** Maya Salomonsson, Joakim S. Dahlin, Johanna Ungerstedt, Jenny Hallgren

**Affiliations:** ^1^Department of Medical Biochemistry and Microbiology, BMC, Uppsala University, Uppsala, Sweden; ^2^Department of Medicine, Huddinge, Karolinska Institutet, and PO Hematology, Karolinska University Hospital, Stockholm, Sweden

**Keywords:** mast cells, mast cell progenitors, chemokine receptors, mouse, human

## Abstract

Mast cells are powerful immune cells found predominately in barrier tissues. They play an important role in immune surveillance and act as effector cells in allergic reactions. Mast cells develop from mast cell progenitors (MCp), which migrate to the peripheral tissues via the blood circulation. Presumably, the homing of MCp to the peripheral sites and localization is regulated by chemotactic signals. Due to the scarce abundance of these cells, chemotactic receptors have not been previously characterized on primary MCp. Here, mRNA transcripts for CCR1 and CX_3_CR1 were identified in mouse bone marrow and lung MCp in a gene expression screen of chemotactic receptors. However, surface expression of CCR1 was only found in the bone marrow MCp. Flow cytometry-based screening identified distinct surface expression of CCR5 by mouse peritoneal mast cells and MCp, while surface expression of CXCR2-5, CX_3_CR1, CCR1-3, CCR6-7, and CCR9 was not detected. Low surface expression of CCR5 was detected in mouse MCp in the bone marrow, spleen, and lung. To translate the findings to human, blood and bone marrow MCp from healthy donors were analyzed for possible CCR1 and CCR5 expression. Human MCp showed distinct surface expression of both CCR1 and CCR5. The expression levels of these chemokine receptors were higher in human bone marrow MCp than in the peripheral blood, suggesting that CCR1 and CCR5 may mediate retention in the bone marrow. In conclusion, mouse and human MCp show differential expression of CCR1 and CCR5 depending on their localization.

## Introduction

Mast cells are rare and long-lived tissue-resident immune cells, which play an important role in innate immunity as first-responders in the peripheral tissues, and as potent effector cells by Fc-receptor-mediated activation. In embryonic development, the first mast cells that populate the tissues derive from progenitors in the yolk sac (1). In adult mice, mast cells seem to have a dual origin with long-lived connective tissue type mast cells mainly originating from yolk sac-derived mast cell progenitors (MCp), and mucosal mast cells developing from bone marrow-derived MCp that originate from fetal hematopoietic stem cells (2). In humans, a MCp population is present in peripheral blood and bone marrow (3, 4). Mouse and human MCp are extremely rare, corresponding to ~0.005% of blood mononuclear cells (5). They have a progenitor type morphology with emerging granular structures in the scarce cytoplasm. In peripheral tissues, mouse MCp are distinguished from mature mast cells by the difference in side scatter (SSC) properties and expression of integrin β7 (6), an integrin that also human blood MCp express (3).

During homeostatic conditions, MCp home to peripheral tissues in very low numbers. However, mast cells accumulate in the affected tissues during inflammation. For example, mast cells accumulate in human coronary arteries, increase upon plaque progression, and their number correlates with the number of micro vessels within the plaque (7). Mast cells also accumulate at specific sites in the lung in patients with allergic asthma, and mouse models reveal that the accumulation depends on the recruitment and maturation of MCp originating in the bone marrow (5). The chemokine receptors CXCR2 and CCR2 regulate antigen-induced recruitment of MCp to the lung in an experimental asthma model (8, 9). Notably, bone marrow-chimera experiments revealed that the expression of CXCR2 is required on stromal cells whereas the expression of CCR2 is required on both stromal and bone marrow-derived cells for an intact antigen-induced recruitment of MCp to the lung. The chemotactic signal(s) directly involved in homing as well as for inflammation-induced recruitment of MCp to the lung *in vivo* are still unknown.

Several studies have investigated the chemotactic responses and expression of chemokine receptors of mouse and human *in vitro*-derived mast cells. Mouse bone marrow-derived mast cells (BMMC) express CCR1-3, and CCR5 mRNA and demonstrate surface expression of CCR3 (8, 10). Stimulation with SCF or IgE-crosslinking upregulated these chemokine receptors, and their respective ligands induced directed migration of the BMMCs (11). Expression of CXCR-family chemokine receptors (CXCR2-4) have also been shown in isolated human lung mast cells conditioned *in vitro* and human cord blood-derived mast cells (12, 13). Moreover, leukotrienes and prostaglandins have also been demonstrated to promote migration of BMMCs via BTL1 and EP3, respectively, and intravenously injected BMMCs accumulated in the skin after leukotriene B_4_ or prostaglandin E_2_ was injected intradermally (14, 15).

Earlier studies have given insights to potential migratory mechanism for the movement of mature mast cells. However, mature mast cells are absent in the blood. Instead, circulating MCp populate the tissues. The aim of our study was therefore to determine which chemokine receptors that are expressed by primary MCp. A gene expression analysis of chemotactic receptors demonstrated transcripts of CCR1 and CX_3_CR1 in mouse lung and bone marrow MCp. CCR1 protein was detected on the surface of MCp in the bone marrow but not in the lung, whereas CX_3_CR1 surface expression was not detected at either site. A flow cytometry-based screen of peritoneal MCp and mature mast cells showed distinct surface expression of CCR5, whereas MCp from bone marrow, spleen and lung had low CCR5 surface expression. Finally, we report that human MCp from bone marrow and blood show surface expression of both CCR1 and CCR5, and that the expression of these receptors is highest in the bone marrow. In conclusion, MCp in different compartments of the body show localization-specific expression of CCR1 and CCR5.

## Materials and Methods

### Mice

Mice were bred and maintained in the animal facility at the National Veterinary Institute (SVA), Uppsala, Sweden. Wild type BALB/c were originally obtained from Bommice (Ry, Denmark). At least six weeks old female mice were used for most of the experiments, except for the experiments involving influenza infection were only male mice were used.

### Extraction of Mouse Cells

Mice were euthanized with isoflurane. The peritoneal cells were extracted by flushing the peritoneal cavity with 4 ml staining buffer [2% Fetal Calf Serum (Sigma-Aldrich, St. Louis, MO, USA) in PBS, pH 7.4]. Spleen and thymus were grinded using a nylon mesh to release the cells. Bone marrow cells were obtained from tibiae and femurs by flushing out the cells by a syringe with staining buffer. The red blood cells in the cell suspensions were lysed with lysis buffer (150 mM NH4Cl, 9.5 mM NaHCO3 or 10 mM KH CO3, 1.2 mM EDTA, pH 7.3-7.4). The lungs were flushed with 10 ml PBS through the right ventricle of the heart to remove blood before they were mechanically and enzymatically dissociated using the gentleMACS Octo Dissociator and Lung Disassociation Kit (Miltenyi Biotec, Bergisch Gladbach, Germany) into single cell suspensions in accordance to the manufacture's protocol. Tissue residues were removed using 44% Percoll (Sigma-Aldrich, St. Louis, MO, USA). The cell numbers were determined by manual counting using trypan blue exclusion in a hemocytometer.

### Influenza A Virus Infection

Mice were anesthetized with isoflurane and infected intranasally with 4 x 10^4^ tissue culture infectious dose 50 (TCID_50_) of the H1N1 influenza A/Puerto Rico/8/34 strain. Control mice were administered an equal intranasal volume of sterile PBS (pH 7.4). The mice were weighted before the administration of the virus and the weight was determined regularly until the termination of the experiments. Mice that lost ≥15% of their initial weight before the endpoint were euthanized and excluded from the study.

### Human Blood and Bone Marrow

All five donors that participated in the study gave their written, informed consent. The healthy subjects donated both blood and bone marrow samples at one single hospital visit. Stockholm Regional Ethics committee, Dnr 2015/1914-31/1 approved the study protocol, which was conducted according to the Declaration of Helsinki. The human bone marrow was collected into 15 ml falcon tubes with RPMI media containing heparin. Blood samples from the same individuals were collected by venipuncture into EDTA-treated tubes (10 ml; BD Vacutainer, BD Bioscience, Franklin Lakes, NJ, USA), or from buffy coats obtained from anonymized blood donors at Uppsala University hospital. The mononuclear cell populations were enriched using Ficoll-Paque Premium (ρ = 1.076 g/ml) (GE Healthcare, Little Chalfont, UK) in SepMate™-50 tubes (Stemcell Technologies, Vancouver, Canada). Platelets were removed by centrifugation (2 × 200g, 10 min).

### Flow Cytometry

The following antibodies were used for detection of mouse MCp and mast cells in peritoneal lavage: CD117-PE-Cy7 (2B8), CD16/32-V450 Horizon (2.4G2) or CD16/32-Brilliant Violet 421 (2.4G2), integrin β7-FITC (FIB504), and PE-Cy5 conjugated lineage markers CD3 (17A2), CD4 (GK1.5), CD8b (eBioH35-17.2), B220 (RA3-6B2), Gr-1 (RB6-8C5), CD11b (M1/70), CD19 (eBio1D3), and TER119 (TER-119). For detection of chemokine receptors phycoerythrin (PE)-conjugated antibodies for the different chemokine receptors were used (the specific antibodies are listed in **Table 3**). MCp in the lung, spleen and bone marrow were detected with the same antibody panel as above was used with the addition of antibodies recognizing CD45 [CD45-AlexaFluor700 (30-F11)] and T1/ST2 (T1/ST2-Brilliant Violet 421 (DIH9) or biotinylated T1/ST2 (DJ8) followed by incubation with streptavidin-APC). For re-analysis of the cultured bone marrow MCp, DX5-V450 (DX5) and FcεRI-PE (MAR-1) was used. Unlabeled CD16/32 (2.4G2) was used as an Fc-block in experiments in which fluorochrome-labeled CD16/32 was not used to distinguish a cell population. To facilitate detection of cell surface expression of chemokine receptors (16), the incubation of antibodies was performed in dark at 37°C. Fluorescence minus one (FMO) controls with the PE conjugated isotype controls for each chemokine receptor added were used as controls. Enriched mononuclear cells from blood or bone marrow were incubated in staining buffer with the following fluorescence-labeled antibodies to analyze human MCp: CD4 (RPA-T4), CD8 (RPA-T8), CD13 (WM15), CD14 (M5E2), CD19 (HIB19), CD34 (581), CD117 (104D2), and FcεRI (AER-37). The antibodies listed in **Table 3**, were added to the above-mentioned panel to analyze chemokine receptor expression. Due to the limited number of cells from human blood and bone marrow a modified FMO control were used (fluorescence minus two; FMT) to control for the chemokine receptor expression. In the FMT control, both chemokine receptors antibodies were excluded in the same control sample. This method was verified using buffy coats from the local blood donor center. All antibodies were obtained from BD Biosciences, BioLegend, eBioscience, R&D Systems or MD bioproducts (Supplementary Table 1). Flow cytometry was performed on an LSR II, LSR Fortessa or the FACSAria III (BD Biosciences). FlowJo software version 9.8 and 10.3.0 was used to analyze the data and to prepare the graphs.

### Cell Culture

The sorted mouse bone marrow cells were cultured in Iscove's Modified Dulbecco's Medium supplemented with 20% heat-inactivated fetal calf serum, 2 mM L-glutamine, 10 mM HEPES, 100 U/mL penicillin, 100 μg/mL streptomycin, 10 μg/mL gentamicin, 1 × MEM nonessential amino acids, 50 μM 2-mercaptoethanol, 1 mM sodium pyruvate. The medium was supplemented with a myelo-erythroid cytokine cocktail consisting of 20 ng/mL each of recombinant mouse IL-3, IL-6, IL-7, IL-9, IL-11, thrombopoietin, stem cell factor, granulocyte-macrophage colony-stimulating factor purchased from Peprotech (Rocky Hill, NJ), IL-5 and erythropoietin from R&D Systems (Minneapolis, MN). The medium and the supplements were from Sigma-Aldrich (St. Louis, MO). The cells were cultured at 37°C with 5% CO_2_.

### qPCR

The cells (100–200) were sorted by fluorescence activated cell sorting (FACS) directly into RLT lysis buffer (Qiagen, Hilden, Germany), placed into cryotubes, snap frozen in liquid nitrogen, and stored in liquid nitrogen or a −80°C freezer until RNA extraction. Extraction of RNA was preformed using the RNeasy Mini Kit (Qiagen). Synthesis of cDNA and pre-amplification was done with Whole Transcriptome Amplification Kit 1 (Sigma-Aldrich) in combination with JumpStart AccuTaq LA DNA Polymerase (Sigma-Aldrich). The primers were designed using Primer 3 software, and produced by LGC Biosearch Technologies (Risskov, Denmark). The primers are listed in Table 1. For detection, PowerUp SYBR Green Master Mix (Thermo Fisher Scientific, Waltham, MA, USA) was used and the samples were analyzed with CFX96 Touch Real-Time Detection System (Bio-Rad, Hercules, CA, USA using the CFX Maestro software (Bio-Rad). Ribosomal protein L30 (RPL30), Polyubiquitin-C (UBC) and Glyceraldehyde 3-phosphate dehydrogenase (GAPDH) were used as endogenous controls. To verify the primers, ~3 × 10^4^ BMMCs (9 weeks old) from a C57BL/6 mouse which were cultured in 30% WEHI-3B-conditioned media, 1% Penicillin-Streptomycin (PEST; SVA, Uppsala, Sweden), 1% L-glutamine (SVA, Uppsala, Sweden), 10% Fetal bovine serum (FBS; Invitrogen, Carlsbad, CA, USA), Dulbecco's Modified Eagle's Medium (DMEM; Sigma-Aldrich, St. Louis, MO, USA) were used. Cells were lysed with RLT lysis buffer (Qiagen) and extraction of RNA was preformed using the RNeasy Mini Kit (Qiagen). cDNA was synthesized using the Iscript cDNA Synthesis Kit (Bio-Rad, Hercules, CA, USA).

**Table 1:** Primers used for detection of chemotactic receptors and endogenous controls.

**Target gene**	**Forward sequence (5′-3′)**	**Reverse sequence (5′-3′)**
BLT1	TCC TCC ACC ATT CCT GAG TC	ATC CTG TCT CTC TGC CCT GA
EP3	TGC CAT TAA ACA CAC CGA GA	ACA AAG GTT CTG AGG CTG GA
CXCR2	ATG CCC TCT ATT CTG CCA GAT	GTG CTC CGG TTG TAT AAG ATG AC
CXCR3	TAC CTT GAG GTT AGT GAA CGT CA	CGC TCT CGT TTT CCC CAT AAT C
CXCR5	CCA AGC AGA AAG CTG AAA CC	ACT TTT CCA CTG GGC CTC TT
CX_3_CR1	CTC ACC ATG TCC ACC TCC TT	CGA GGA CCA CCA ACA GAT TT
CCR1	AGG GCC CGA ACT GTT ACT TT	TTC CAC TGC TTC AGG CTC TT
CCR2	TTT GCA ACT GCC TCT TTC CT	CTT CTG TCC CTG CTT CAT CC
CCR3	TTT CCT GCA GTC CTC GCT AT	ATA AGA CGG ATG GCC TTG TG
CCR4	TTC CAA AGA TGA ATG CCA CA	CCC AAC AAG AAG ACC AAG GA
CCR5	ATG GAT TTT CAA GGG TCA GTT CC	CTG AGC CGC AAT TTG TTT CAC
CCR6	GTT GAA CAT GGC CAT CAC AG	TAC CGG TCC ATG CTG ATA CA
CCR7	GTG TGC TTC TGC CAA GAT GA	CCA CGA AGC AGA TGA CAG AA
CCR8	GCA GTC TTT GAG GTG GAA GC	TTG AAT GGG ACC CAG AAG AG
CCR9	GCT GGT TGC ACA GAG AAA CA	ACC CTG GTT GGG AAT TAA CC
RPL30	CCG CAA AGA AGA CGA AAA AG	GGA CAG TTG TTG GCA AGG AT
UBC	AGC CCA GTG TTA CCA CCA AG	TCA AAG TGC AAT GAA ACT TGT TA
GAPDH	CTC CCA CTC TTC CAC CTT CG	CCA CCA CCC TGT TGC TGT AG

### Statistics

Statistical differences between groups were assessed using Student's *t*-test. All graphs were prepared and statistics calculated using GraphPad Prism 5.0f (GraphPad software Inc., San Diego, CA). A *p*-value of <0.05 was considered significant.

## Results

### Cell-Fate Assays Reveal a Population of BALB/c Bone Marrow Cells With Mast Cell-Forming Potential

**Figure 1:**
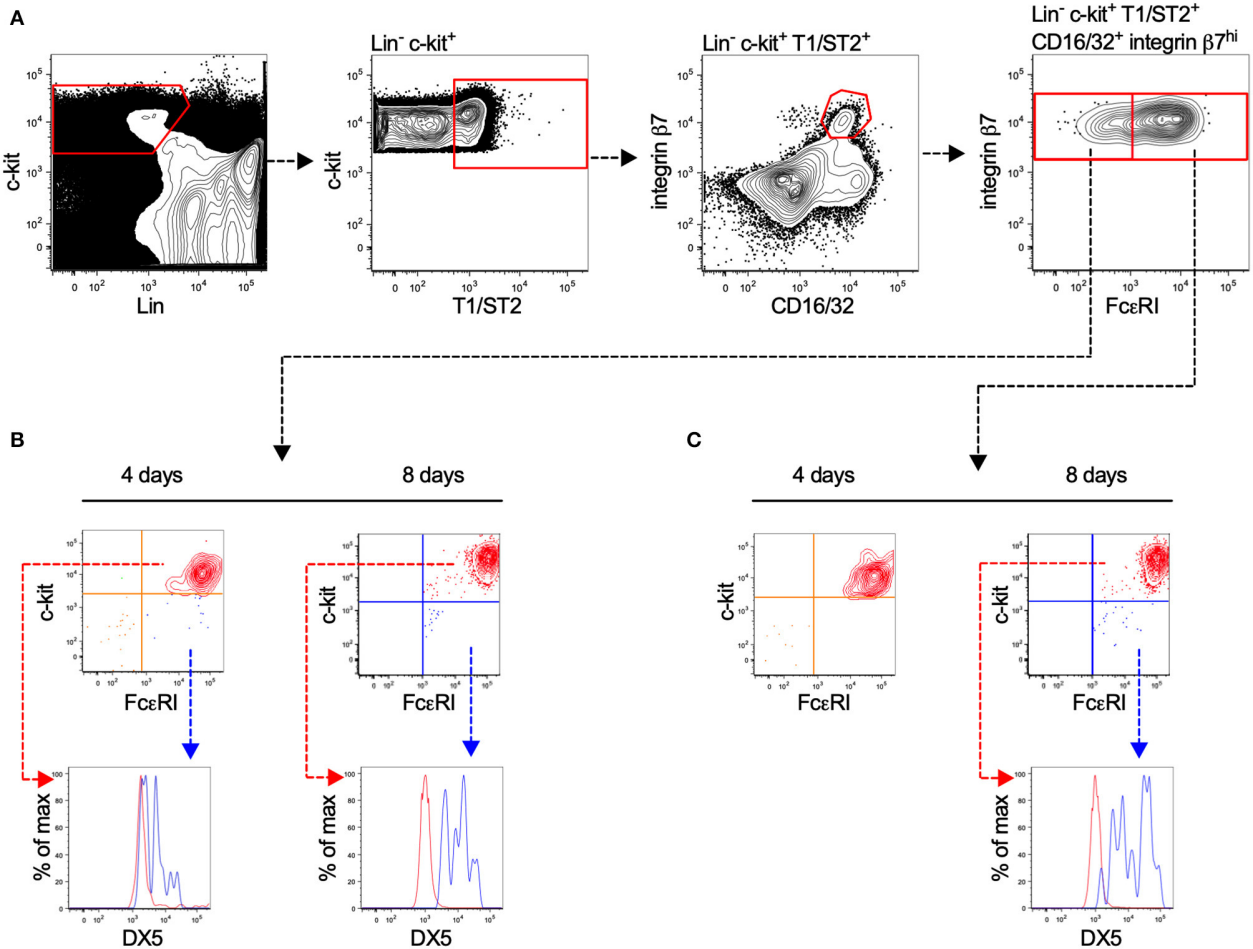
Lin^−^ c-kit^+^ T1/ST2^+^ integrin β7^hi^ CD16/32^+^ bone marrow progenitors demonstrate mast cell-forming potential independent of FcεRI expression. **(A)** Bone marrow cells from naïve BALB/c mice were stained with fluorescently-conjugated antibodies and analyzed by flow cytometry. The Lin^−^ c-kit^+^ T1/ST2^+^ CD16/32^+^ integrin β7^hi^ cells were gated and divided into FcεRI^+^ and FcεRI^−^ populations. The cells were FACS-sorted in to wells containing media supplemented with a myelo-erythroid cytokine cocktail. **(B,C)** After four and eight days of culture, the progenies were analyzed for their surface expression of FcεRI, c-kit, and DX5. The graphs shown are representative of two independent experiments analyzed days four and eight.

Committed MCp have previously been identified in the bone marrow of C57BL/6 mice (17), but not in BALB/c mice. Here, we isolated prospective populations of bone marrow MCp based on the earlier identified forms of MCp in the blood circulation of BALB/c mice, which either have or lack surface expression of FcεRI (18). Lineage (Lin^−^) c-kit^+^ T1/ST2^+^ CD16/32^+^ integrin β7^hi^ bone marrow cells lacking or expressing FcεRI were sorted by FACS, and cultured in a myelo-erythroid cytokine cocktail (Figure 1A). After four or eight days of culture, the progenies were analyzed by flow cytometry for their surface expression of FcεRI, c-kit, and DX5, also referred to as integrin α2 or CD49b (a basophil marker). Both isolated populations displayed progenies with a similar percentage of c-kit^+^ FcεRI^+^ cells, i.e., 74–79% were double positive for c-kit and FcεRI after four days and 99–100% were double positive after eight days (Figures 1B,C). A minor fraction of the *in vitro*-differentiated cells expressed FcεRI, DX5 and lacked c-kit expression (Figures 1B,C), suggesting a little residual basophil potential in the progenitor populations. We conclude that the majority of the Lin^−^ c-kit^+^ T1/ST2^+^ CD16/32^+^ integrin β7^hi^ bone marrow cells, independent of their FcεRI expression, develop into mast cells. Hence, Lin^−^ c-kit^+^ T1/ST2^+^ CD16/32^+^ integrin β7^hi^ bone marrow MCp were used in the following experiments.

### Mouse Bone Marrow MCp Express mRNA Transcripts and Surface Expression of CCR1

**Figure 2:**
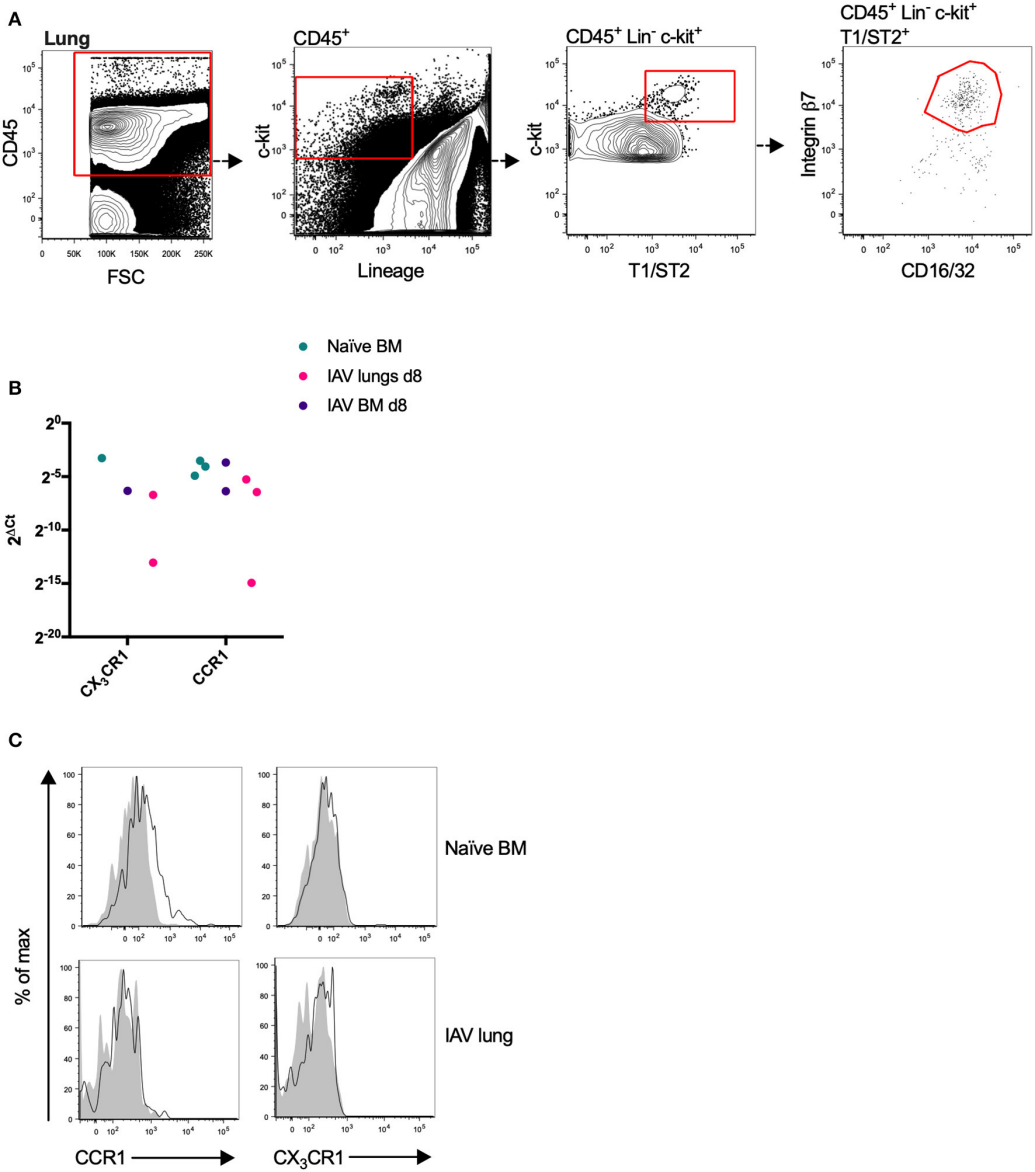
Mouse MCp in the bone marrow express CCR1 transcripts and protein. **(A)** Lung MCp were identified as CD45^+^ Lin^−^ c-kit^+^ T1/ST2^+^ integrin β7^hi^ CD16/32^+^ cells. **(B)** MCp from naïve or influenza-infected (IAV) bone marrow (BM) or lungs were FACS-sorted, lysed and analyzed by qPCR for the mRNA expression of chemotactic receptors. CCR1 and CX_3_CR1 mRNA were detected in the majority of the samples. The relative expression using RPL30 as the endogenous control is shown. All detected values from three independent experiments are shown. **(C)** Representative histograms from three experiments aimed at determining CCR1 and CX_3_CR1 surface expression by bone marrow MCp from naïve mice or lung MCp from influenza (IAV) infected mice, compared with FMO control including isotype control staining.

To explore the expression pattern of chemotactic receptors on MCp from the bone marrow and lung, a qPCR-based expression screen was used. Approximately 100–200 MCp from bone marrow of naïve mice, and bone marrow and lung from influenza-infected mice were FACS-sorted (Figures 1A, 2A) into lysis buffer, and subjected to qPCR following cDNA pre-amplification. We have previously demonstrated that influenza-infection in mice triggers the recruitment of MCp to the lung (19). To allow the isolation of MCp, the lungs were harvested eight days post-infection with influenza virus. Before the screening of mRNA for chemotactic receptors, all the primers in Table 1, were verified to give a positive signal when BMMCs were analyzed by qPCR (data not shown).

CCR1 and CX_3_CR1 mRNA transcripts were detected in at least one MCp sample out of three from all three organs (Figure 2B, Table 2). Gene expression of the other chemokine receptors were not detected or not consistently found in the MCp samples (Table 2). Bone marrow cells from naïve mice and lung cells from influenza-infected mice were analyzed by flow cytometry to test whether CCR1 and CX_3_CR1 could be detected at the protein level. The MCp in the bone marrow were defined as Lin^−^ c-kit^+^ T1/ST2^+^ CD16/32^+^ integrin β7^hi^ cells (Figure 1A) and the lung MCp were identified as CD45^+^ Lin^−^ c-kit^hi^ T1/ST2^+^ integrin β7^hi^ CD16/32^+^ cells, similar to Zarnegar et al. (19) (Figure 2A). A consistent positive signal for CCR1 relative to the signal from the FMO with the appropriate isotype control staining was found on MCp from naïve bone marrow, indicating surface expression of CCR1 (Figure 2C). However, the bone marrow MCp lacked surface expression of CX_3_CR1 (Figure 2C). The analyses of lung MCp were performed eight days post-infection when the lung MCp have expanded due to the influenza-induced recruitment (19). Nevertheless, surface expression of CCR1 or CX_3_CR1 could not be detected on the MCp from influenza-infected lungs (Figure 2C). Transcripts for BLT1 and EP3 could be detected in some of the MCp samples (Table 2). However, as commercial antibodies designed for flow cytometry to check expression of these receptors are lacking, we were unable to verify expression of these receptors in MCp on the protein level.

**Table 2:** The mRNA expression of chemotactic receptors on sorted MCp from naïve bone marrow, influenza infected (IAV) bone marrow or lung.

	**Naïve bone marrow**	**IAV bone marrow**	**IAV lung**
BLT1	36, –, 30 (2/3)	–, 29, 34, (2/3)	–, –, –, (0/3)
EP3	–, –, –, (0/3)	–, 31, –, (1/3)	32, 33, –, (2/3)
CXCR2	–, –, –, (0/3)	–, 32, 35, (2/3)	33, 33, –, (2/3)
CXCR3	–, –, –, (0/3)	–, 37, –, (1/3)	38, 36, – (2/3)
CXCR5	–, –, –, (0/3)	–, 31, –, (1/3)	33, 33, –, (2/3)
CX_3_CR1	26, 26, 36 (3/3)	–, 31, – (1/3)	32, 26, – (2/3)
CCR1	26, 28, 28 (3/3)	29, 28, 35 (3/3)	33, 35, 25 (3/3)
CCR2	–, –, –, (0/3)	35, 32, 34, (3/3)	34, 32, –, (2/3)
CCR3	–, 28, –, (1/3)	–, 31, –, (1/3)	34, 32, –, (2/3)
CCR4	–, –, –, (0/3)	–, 33, –, (1/3)	35, 34, –, (2/3)
CCR5	–, –, –, (0/3)	–, 32, –, (1/3)	34, 34, –, (2/3)
CCR6	–, –, –, (0/3)	–, 32, –, (1/3)	–, 34, –, (1/3)
CCR7	–, –, –, (0/3)	–, 32, –, (1/3)	33, 33, –, (2/3)
CCR8	–, –, –, (0/3)	–, 31, 34, (2/3)	32, 33, –, (2/3)
CCR9	–, –, –, (0/3)	–, 32, 35, (2/3)	34, 33, –, (2/3)
RPL30	23, 24, 23 (3/3)	23, 25, 20 (3/3)	20, 19, 20 (3/3)
UBC	29, –, –, (1/3)	–, 32, 34, (2/3)	26, 33, – (2/3)
GAPDH	–, 30, 26 (2/3)	27, 26, 27, (3/3)	25, 24, 25 (3/3)

*The number of PCR cycles required to reach the threshold is shown for each experiment and target. The number of experiments with a detected signal is shown in the parenthesis. RPL30, UBC and GAPDH were used as endogenous controls*.

*The number in parenthesis indicate number of experiments with signal for the specific receptor. –, no signal*.

### Mouse Peritoneal MCp Show Surface Expression of CCR5 at Homeostatic Conditions

**Figure 3:**
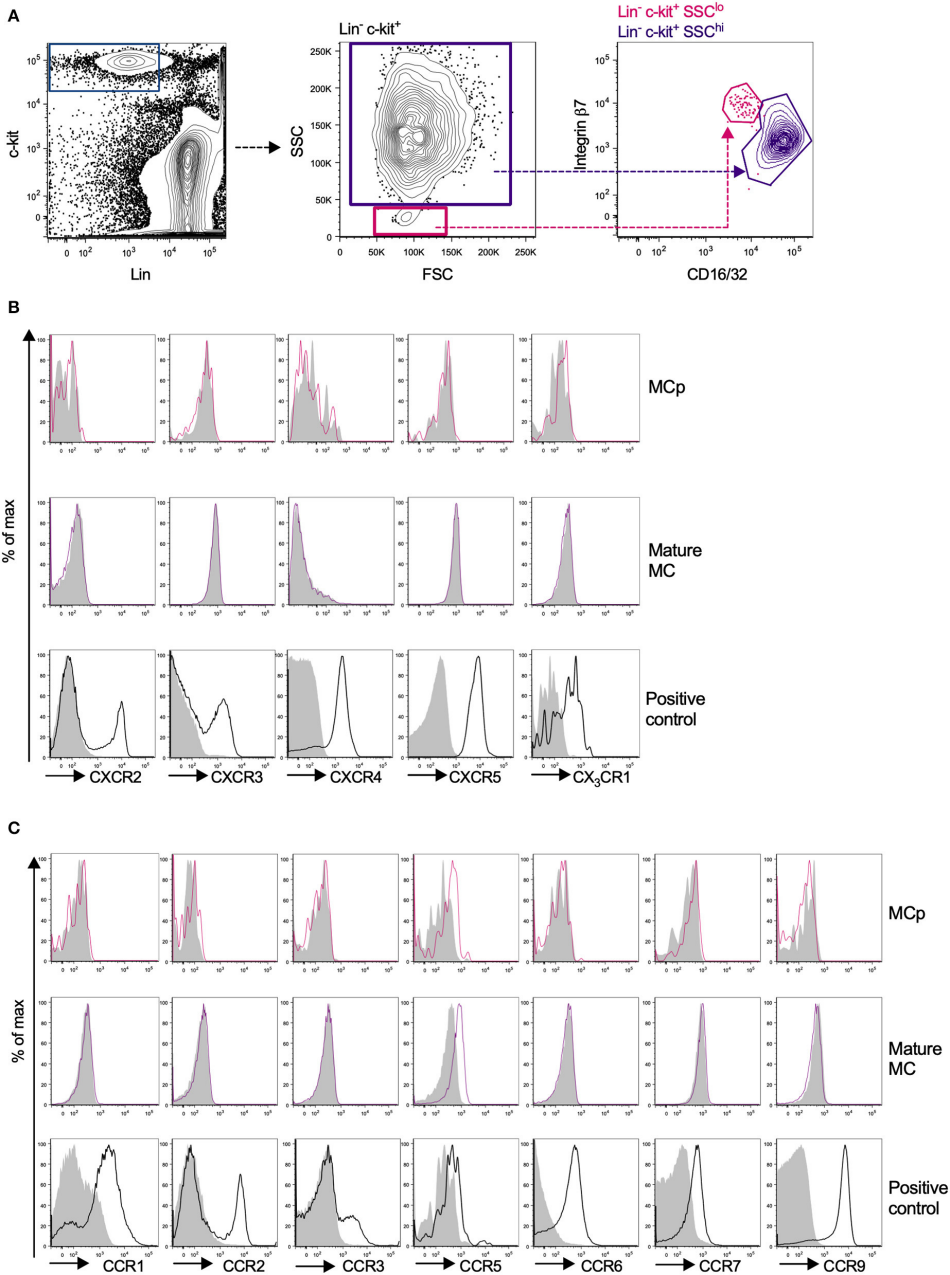
Mouse peritoneal MCp demonstrate CCR5 surface expression but lack expression of other CCR-family, CXCR-family receptors and CX_3_CR1. Peritoneal cells from naïve BALB/c mice were stained by fluorescently-labeled antibodies and analyzed by flow cytometry. **(A)** MCp were identified as Lin^−^ c-kit^+^ SSC^lo^ integrin β7^hi^ CD16/32^int^ cells and mast cells as Lin^−^ c-kit^+^ SSC^hi^ integrin β7^int^ CD16/32^hi^ cells. **(B)** Representative histograms for the analyses of CXCR2-5 and CX_3_CR1 surface expression on MCp, mast cells (MC) and positive control cells. **(C)** Representative histograms for the analyses of CCR1-3, CCR5-7, and CCR9 surface expression on MCp, MC and positive control cells. The positive controls for each chemokine receptor are described in Table 3. Pooled cells from three-six mice were used for each individual experiment. Each chemokine receptor was analyzed in two-three separate experiments.

Due to the relatively high abundance of mast cells in the peritoneum, chemokine receptor expression on mouse peritoneal MCp and mast cells was assessed by flow cytometry. MCp were identified as Lin^−^ c-kit^+^ SSC^lo^ integrin β7^hi^ CD16/32^int^ cells and mature mast cells as Lin^−^ c-kit^+^ SSC^hi^ integrin β7^int^ CD16/32^hi^ peritoneal cells (Figure 3A). Expression analysis of FcεRI and T1/ST2 verified that the populations were identical to those we previously described (6) (Supplementary Figure 1A). The surface expression of the chemokine receptors for which a positive control cell population could be identified was determined by flow cytometry (Table 3). Peritoneal MCp and mature mast cells lacked expression of CXCR2-5 and CX_3_CR1 (Figure 3B). They also lacked surface expression of CCR1-3, CCR6, CCR7, and CCR9 (Figure 3C). However, both peritoneal mast cells and MCp demonstrated distinct surface expression of the chemokine receptor CCR5 (Figure 3C, Table 4).

**Table 3:** Description of chemokine receptor detection antibody.

	**Antibody target**	**Clone**	**Positive control**
Mouse	CX_3_CR1	528728	CD115^+^ Gr-1^−^ CD16/32^hi^ splenocytes
	CXCR2	242216	CD11b^+^ splenocytes
	CXCR3	CXCR3-173	CD3^+^ splenocytes
	CXCR4	2B11/CXCR4	CD4^+^ cells from thymus
	CXCR5	SPRCL5	CD19^+^ cells from peritoneal cavity
	CCR1	643854	CD115^+^ Gr-1^−^ splenocytes
	CCR2	475301	CD11b^+^ splenocytes
	CCR3	J073E5	Gr-1^+^ splenocytes
	CCR5	HM-CCR5 (7A4)	DX-5^+^ CD11b^+^ CD4^−^ CD8^−^ splenocytes
	CCR6	29-2L17	B220^+^ splenocytes
	CCR7	4B12	CD4^+^ splenocytes
	CCR9	eBioCW-1.2	CD4^+^ cells from thymus
Human	CCR5	3A9	CD14^+^ monocytes from bone marrow and blood
	CCR1	53504	CD14^+^ monocytes from bone marrow and blood

*Clone of detection antibody used for detection. Description of which cells and antibodies that were used to identify positive controls for each chemokine receptor*.

**Table 4:** CCR5 expression expressed as gMFI on MCp and mast cells (MC) in mouse peritoneum (PT), lung, spleen and bone marrow (BM).

	**MCp**				**MC**	
	**Isotype**	**Naive**	**PBS**	**IAV**	**Isotype**	**Naive**
PT	167	265	–	–	530	616
	182	397	–	–	404	528
	163	357	–	–	337	754
Lung	28.7	137	–	–	–	–
	391	–	1359	730	–	–
	45.3	–	101.3^(a)^	86.3	–	–
	29.8	–	44.3^(a)^	64.7	–	–
	72.1	–	85.5^(a)^	105.7	–	–
Spleen	113	157	–	–	–	–
	57.3	72.6	–	–	–	–
	53.7	86.2	–	–	–	–
BM	84.6	117	–	–	–	–
	37.7	55.9	–	–	–	–
	45.1	106	–	–	–	–

*Each row represents one experiment*.

(a) *Mean from three individually analyzed mice*.

### Mouse MCp in the Bone Marrow, Spleen and Lung Demonstrate Low Surface Expression of CCR5

**Figure 4:**
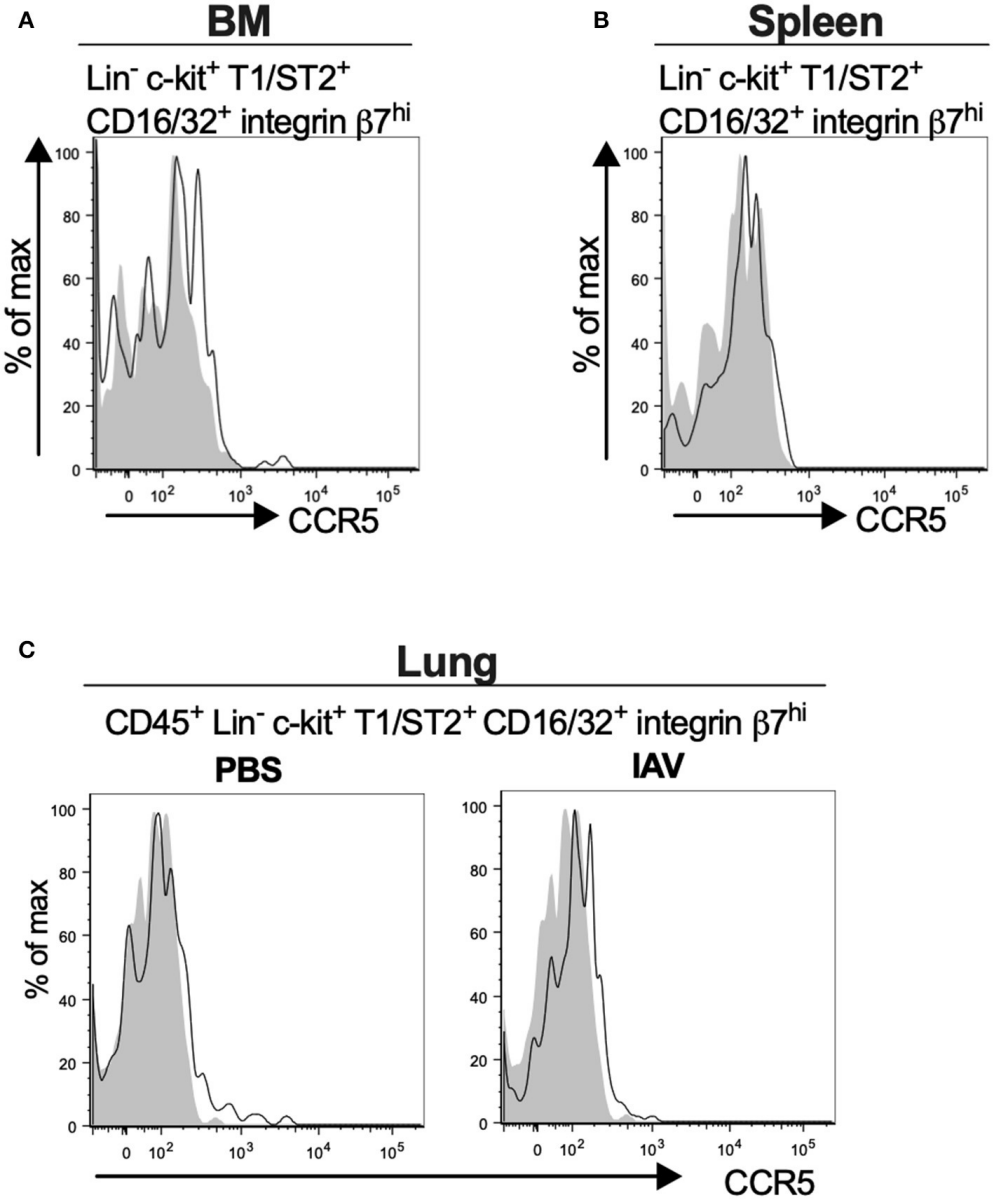
Mouse MCp in the bone marrow, spleen and lung demonstrate surface expression of CCR5. **(A,B)** Bone marrow (BM) and spleen cells from naïve BALB/c mice were stained by fluorescently-labeled antibodies and analyzed by flow cytometry. **(A)** Representative histograms from three independent experiments demonstrating surface expression of CCR5 by BM MCp **(B)**. Representative histograms of three independent experiments demonstrating surface expression of CCR5 by spleen MCp. **(C)** Isolated lung cells from PBS-injected or influenza-infected (IAV) mice were stained with fluorescently-conjugated antibodies and analyzed by flow cytometry. Representative histograms from three experiments with three-nine mice per group per experiment demonstrating surface expression of CCR5 by lung MCp.

Bone marrow, spleen and lung MCp were analyzed by flow cytometry to determine whether MCp from other sites also express CCR5. The Lin^−^ c-kit^+^ T1/ST2^+^ CD16/32^+^ integrin β7^hi^ MCp in naive bone marrow demonstrated a gMFI for CCR5 which was consistently higher than the FMO control containing the appropriate isotype control (Figure 4A, Table 4). Similarly, the signal for CCR5 staining of splenic MCp was consistently slightly increased compared to the control (Figure 4B, Table 4, Supplementary Figure 1B). To determine whether resident or newly recruited lung MCp express CCR5, BALB/c mice were infected with influenza A virus or given PBS as a vehicle and their lungs analyzed by flow cytometry six days post-infection. The lung MCp from influenza infected and PBS-treated mice demonstrated a slight but consistent shift in CCR5 expression (Figure 4C, Table 4). To conclude, MCp in bone marrow, spleen and lung demonstrate surface expression of CCR5.

### Human MCp Demonstrate Surface Expression of CCR1 and CCR5

**Figure 5:**
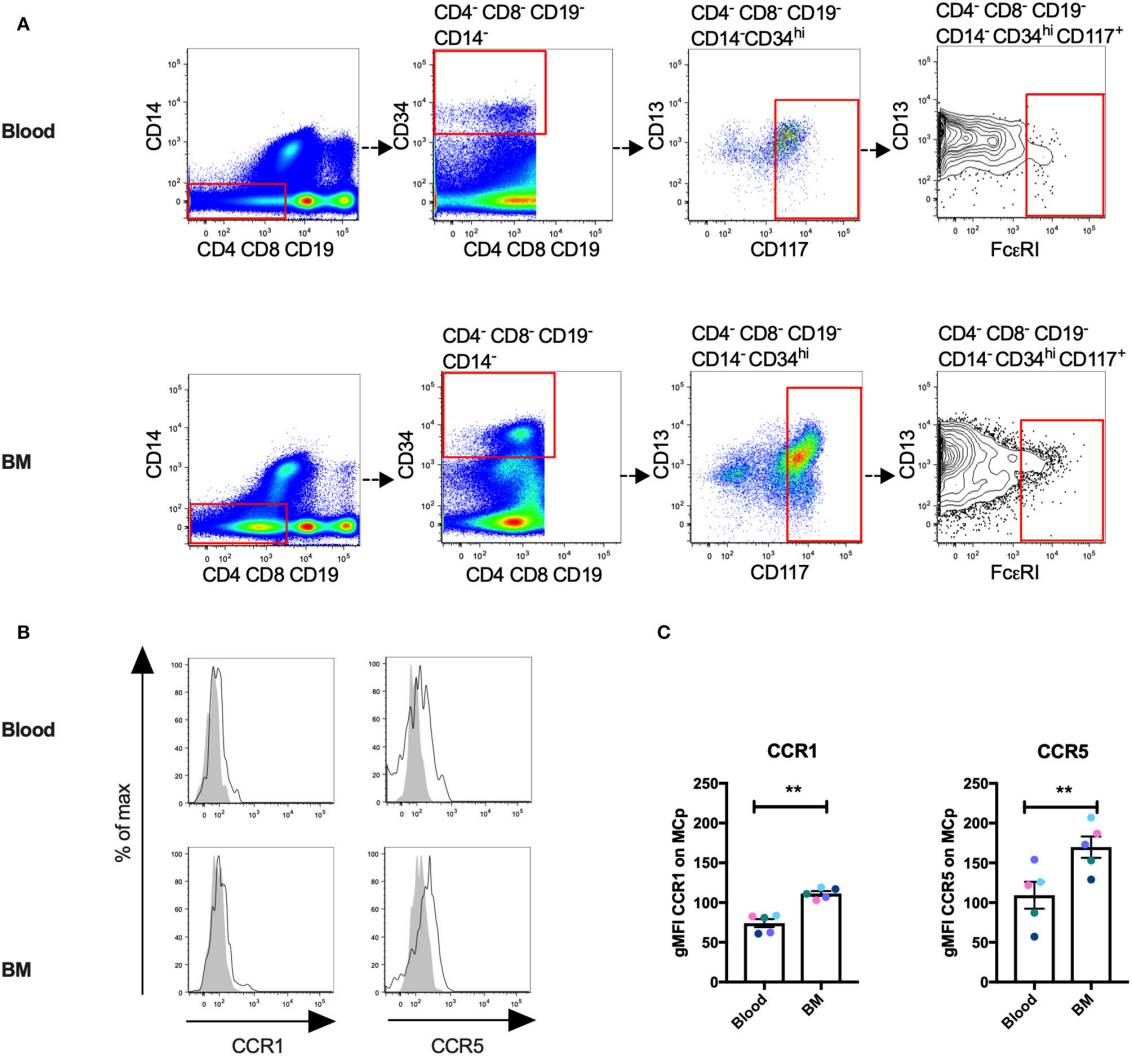
Human MCp in bone marrow and peripheral blood demonstrate surface expression of CCR1 and CCR5. Ficoll-separated cells from bone marrow (BM) and blood of five healthy donors were stained with fluorescently-conjugated antibodies and analyzed by flow cytometry. **(A)** MCp were identified as CD4^−^ CD8^−^ CD19^−^ CD14^−^ CD34^hi^ CD117^+^ FcεRI^+^ cells. **(B)** Representative histograms for CCR1 and CCR5 surface expression on blood and BM MCp. **(C)** The differential expression of CCR1 and CCR5 by MCp from blood and BM were compared and illustrated as a difference in gMFI. Each donor is labeled by a specific color. The bars show the mean ± SEM. ^**^p < 0.01.

We have previously described a rare population of human MCp in the peripheral blood and bone marrow (3, 4). Here, human MCp from blood and bone marrow were analyzed for their possible expression of CCR1 and CCR5. The human MCp were identified as CD4^−^ CD8^−^ CD19^−^ CD14^−^ CD34^hi^ CD117^+^ FcεRI^+^ cells by flow cytometry (Figure 5A). There was a consistent shift and a higher gMFI for both CCR1 and CCR5 compared to the control in the bone marrow and blood MCp (Figure 5B, Table 5). Notably, the expression of CCR1 and CCR5 by bone marrow MCp was higher than the expression by peripheral blood MCp (Figure 5C). We conclude that the human MCp express CCR1 and CCR5, and that the expression change depending on the localization of MCp.

**Table 5:** CCR1 and CCR5 expression (gMFI) by human MCp from peripheral blood or bone marrow (BM).

	**CCR1**		**CCR5**	
	**Control**	**Sample**	**Control**	**Sample**
Blood	23.5	62.4	82.1	154.0
	51.0	83.7	110.0	126
	52.4	82.7	86.6	122
	66.2	81.0	65.7	87.2
	51.8	61.0	58.0	57.2
BM	84.7	107	133	173
	90.5	119	161	207
	75.9	103	124	187
	87.5	111	106	153
	97.3	117	103	129

*Each row represents one experiment*.

## Discussion

The movement and retention of immune cells is regulated by chemotactic molecules binding to an array of different cell surface-associated receptors (20). Until now, expression analyses of chemotactic receptors have been performed on *in vitro*-derived mouse mast cells or in some rare cases on isolated or *in vitro*-differentiated human mast cells with a mature phenotype. The reason for this is apparent as both mast cells and their progenitors are extremely rare cells. As MCp are the cells that move from the bone marrow and give rise to mast cells in the peripheral tissues, the aim of our study was to characterize the expression of chemotactic receptors by MCp. Here, we reveal the gene and protein surface expression pattern of chemotactic receptors on primary MCp.

The mRNA screening of a panel of chemotactic receptors on mouse MCp revealed that transcripts for most receptors could not be detected or were not consistently detected. In contrast, mRNA transcripts of all receptors except CXCR4 were detected in the BMMCs used as positive controls, suggesting that *in vitro*-derived mast cells are poor models of MCp. Strikingly, transcripts for CX_3_CR1 and CCR1 were detected in mouse MCp from all sources investigated. Transcripts of CX_3_CR1 have previously been detected in BMMCs and human dermal foreskin mast cells, and RNA sequencing revealed that yolk sac-derived mast cells isolated from skin of embryos express CX_3_CR1 (1, 21). However, surface expression of CX_3_CR1 could not be detected in MCp from naïve mouse bone marrow or influenza-infected lungs, suggesting that the CX_3_CR1 mRNA is not translated or that post-translational modifications may target the protein for degradation, thus preventing surface expression (22). On the contrary, naïve bone marrow MCp, but not lung MCp from influenza-infected mice, demonstrated a consistent cell surface expression of CCR1. Expression of this chemokine receptor has been detected in conjunctival mast cells by immunohistochemistry, and a role for CCR1 on mast cells *in vivo* was demonstrated in a mouse model of allergic inflammation in the conjunctiva (23). In that study, a CCR1 ligand, CCL3, was required for immediate hypersensitivity reactions *in vivo*. Mice genetically lacking CCL3, and mice that were treated with CCL3-neutralizing antibodies demonstrated attenuated mast cell degranulation. Thus, the detrimental role of CCR1 on mast cells in this setting seems to be related to the enhancement of activation rather than directed migration or retention.

In a second screening approach, flow cytometry was used to screen all chemokine receptors for which a positive control cell population could be identified. CCR5 expression was detected on the peritoneal MCp and mast cells, while they lacked surface expression of all other analyzed chemokine receptors. CCR5 deficient mice have a normal number of MCp in the bone marrow, spleen and intestine (24), indicating that CCR5 is not critical for the retention of MCp in the bone marrow or homing of MCp to these peripheral sites. In agreement with that, surface expression of CCR5 on MCp in the spleen and bone marrow was low, and transcripts for CCR5 was not detected at all in the bone marrow MCp from naïve mice. The CCR5 binding chemokines CCL3-5 are mainly produced upon inflammation, and CCR5 has been shown critical for the recruitment of memory CD8^+^ T cell to the airways during secondary influenza virus infection (25). We could also detect surface expression of CCR5 on lung MCp from influenza-infected mice. However, CCR5 has been shown to be dispensable for antigen-induced recruitment of MCp to the lung (9), and the transmigration mechanisms of MCp in antigen-induced and influenza-associated inflammation seems to be similar (9, 19, 26, 27). Together, this may suggest that CCR5 expression is dispensable for influenza-induced recruitment of MCp to the lung.

Human and mouse MCp share a typical progenitor morphology and the expression of several surface receptors such as integrin β7 and FcεRI (5). In the current study, we demonstrate robust CCR1 and CCR5 surface expression by human bone marrow and peripheral blood MCp. CCR1 and CCR5 are also expressed by various other human myeloid cells including monocytes and immature myeloid cell populations (28, 29). As the human bone marrow and blood samples were obtained from the same healthy donors, the expression level of CCR1 and CCR5 by MCp from the different sites was compared. The human MCp in the bone marrow had a higher expression of both CCR1 and CCR5 than the corresponding cells in the peripheral blood. These data may suggest that CCR1 and CCR5 expression are keeping the human MCp in the bone marrow. In line with this, high levels of CCL3 was found in the bone marrow of patients with multiple myeloma (30), and anti-CCR1 and anti-CCR5 blocking antibodies inhibit the adherence of myeloma cells to bone marrow stroma cells *in vitro* (31). Thus, we speculate that human MCp are retained in the bone marrow by a similar CCR1/CCR5-dependent mechanism and that downregulation of these receptors are necessary for their release into the blood. Given the distinct CCR1 surface expression in naïve mouse bone marrow, it is feasible that CCR1 expression by mouse bone marrow MCp may have a similar function.

In conclusion, our study demonstrates that few chemotactic receptors can be detected on primary mouse MCp. Peritoneal MCp expressed higher surface expression of CCR5 than MCp in other compartments, whereas CCR1 was only expressed by bone marrow MCp on the protein level. In human bone marrow MCp, apparent CCR1 and CCR5 surface expression was detected, while the levels in the blood were lower. Thus, expression of CCR1 and CCR5 is dependent on the localization of the cells. Possibly a high CCR1/CCR5 expression level mediates MCp retention in the bone marrow or peritoneum, respectively. Our study also highlights that detection of mRNA transcripts does not necessarily equal protein expression. Altogether, we provide a comprehensive overview of chemokine receptor expression by primary mouse MCp. Moreover, we confirm protein expression of the two mainly expressed chemokine receptors on the protein level, CCR1 and CCR5, by human MCp.

## Data Availability Statement

All datasets generated for this study are included in the article/Supplementary Material.

## Ethics Statement

The studies involving human participants were reviewed and approved by Stockholm Regional Ethics Committee, Dnr 2015/1914-31/1, which was conducted according to the Declaration of Helsinki. The patients/participants provided their written informed consent to participate in this study. The animal study was reviewed and approved by Stockholm Animal Ethics Committee (N12/14) and Uppsala Animal Ethics Committee (5.8.18-05248/2018).

## Author Contributions

MS and JH designed the study and wrote the paper. JD designed one set of experiments, supervised, and performed those experiments. MS was involved in or performed all experiments, performed the data analysis and the statistical analysis. JH interpreted and supervised the data analysis. JU recruited donors and provided the human samples. All authors read and critically assessed the manuscript.

### Conflict of Interest

The authors declare that the research was conducted in the absence of any commercial or financial relationships that could be construed as a potential conflict of interest.

## Abbreviations

BLT1: leukotriene B_4_ receptor 1
BMMC: bone marrow-derived mast cells
EP3: prostaglandin E receptor 3
FACS: fluorescence activated cell sorting
FMO: fluorescence minus one
gMFI: geometric mean fluorescence intensity
Lin: lineage
MCp: Mast cell progenitors
SSC: side scatter.
