# Supplementary material for: Localization-Specific Expression of CCR1 and CCR5 by Mast Cell Progenitors
Source: Front Immunol. 2020 Feb 26;11:321. doi: 10.3389/fimmu.2020.00321 (PMC7054384; doi:10.3389/fimmu.2020.00321)
Supplement: Supplementary file 1 [file Image_1.pdf]

Supplementary Figure 1

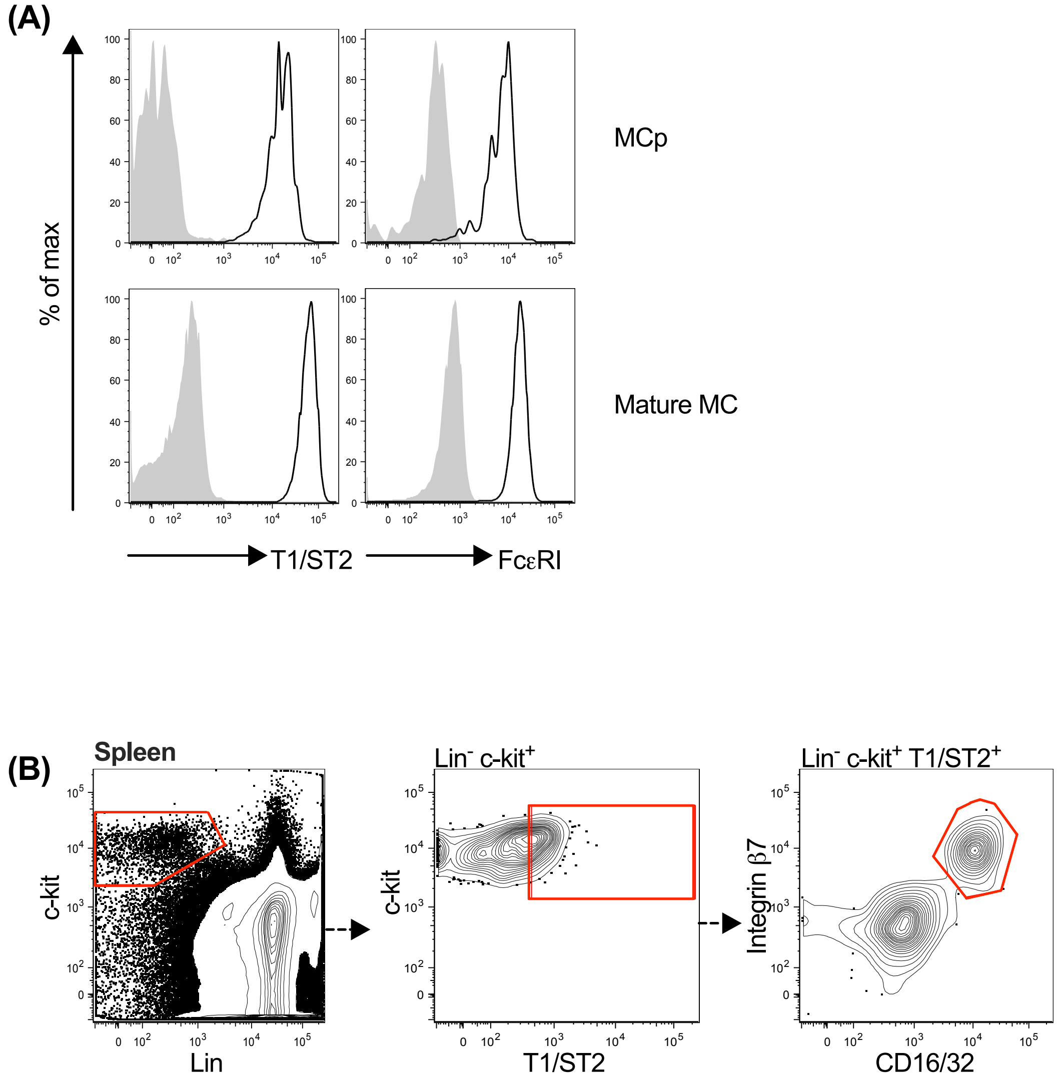

Supplemental Figure 1. *Peritoneal mast cells and their progenitors express T1/ST2 and FcεRI.* (**A**) Peritoneal cells from naïve BALB/c mice were stained by fluorescently-labelled antibodies and analyzed by flow cytometry. MCp were identified as Lin^{-} c-kit^{+} SSC^{lo} integrin β7^{hi} CD16/32^{int} cells and mast cells as Lin^{-} c-kit^{+} SSC^{hi} integrin β7^{int} CD16/32^{hi} cells. Representative histograms demonstrate the surface expression of T1/ST2 and FcεRI by the respective cell population. Pooled cells from 3-6 mice were used in each individual experiment (n=2). (**B**) Spleen cells from naive mice were stained with fluorescently-conjugated antibodies and analyzed by flow cytometry. Representative gating strategy for MCp identified as Lin^{-} c-kit^{+} T1/ST2^{+} integrin β7^{hi} CD16/32^{+} cells.
