# Supplementary material for: Localization-Specific Expression of CCR1 and CCR5 by Mast Cell Progenitors
Source: Front Immunol. 2020 Feb 26;11:321. doi: 10.3389/fimmu.2020.00321 (PMC7054384; doi:10.3389/fimmu.2020.00321)
Supplement: Supplementary file 2 [file Table_1.DOCX]

**Supplementary Table 1.** The clone names and vendors for the antibodies used.

|  | Antibody against | Clone | Company |
| --- | --- | --- | --- |
| MOUSE | CX_3_CR1 | 528728 | R&D Systems |
|  | CXCR2 | 242216 | R&D Systems |
|  | CXCR3 | CXCR3-173 | eBioscience |
|  | CXCR4 | 2B11/CXCR4 | BD Biosciences |
|  | CXCR5 | SPRCL5 | eBioscience |
|  | CCR1 | 643854 | R&D Systems |
|  | CCR2 | 475301 | R&D Systems |
|  | CCR3 | J073E5 | BioLegend |
|  | CCR5 | HM-CCR5 (7A4) | eBioscience |
|  | CCR6 | 29-2L17 | BioLegend |
|  | CCR7 | 4B12 | eBioscience |
|  | CCR9 | eBioCW-1.2 | eBioscience |
|  | CD4 | GK1.5 | eBioscience |
|  | CD8b | eBioH35-17.2 | eBioscience |
|  | CD11b | M1/70 | eBioscience |
|  | CD19 | eBio1D3 | eBioscience |
|  | TER119 | TER-119 | eBioscience |
|  | Gr-1 | RB6-8C5 | eBioscience |
|  | CD117 | 2B8 | eBioscience |
|  | CD16/32 | 2.4G2 | eBioscience |
|  | Integrin β7 | FIB504 | eBioscience |
|  | B220 | RA3-6B2 | eBioscience |
|  | CD115 | AFS98 | eBioscience |
|  | FcεRI | MAR-1 | eBioscience |
|  | CD45 | 30-F11 | eBioscience |
|  | CD3 | 17A2 | BD Biosciences |
|  | DX5 | DX5 | BD Biosciences |
|  | T1/ST2 | DIH9 alt DJ8 | BioLegend alt MD bioproducts |
| HUMAN | CD4 | RPA-T4 | BD Biosciences |
|  | CD8 | RPA-T8 | BD Biosciences |
|  | CD19 | HIB19 | BD Biosciences |
|  | CD13 | WM15 | BD Biosciences |
|  | CD14 | M5E2 | BD Biosciences |
|  | CD34 | 581 | BD Biosciences |
|  | CD117 | 104D2 | BD Biosciences |
|  | FcεRI | AER-37 | eBioscience |
|  | CCR5 | 3A9 | BD Biosciences |
|  | CCR1 | 53504 | BD Biosciences |
